# Serum tissue inhibitor of matrix metalloproteinase-1 levels are associated with mortality in patients with malignant middle cerebral artery infarction

**DOI:** 10.1186/s12883-015-0364-7

**Published:** 2015-07-11

**Authors:** Leonardo Lorente, María M. Martín, Luis Ramos, Juan J. Cáceres, Jordi Solé-Violán, Mónica Argueso, Alejandro Jiménez, Juan M. Borreguero-León, Josune Orbe, José A. Rodríguez, José A. Páramo

**Affiliations:** Intensive Care Unit, Hospital Universitario de Canarias, Ofra, s/n. La Laguna, 38320 Santa Cruz de Tenerife, Spain; Intensive Care Unit, Hospital Universitario Nuestra Señora de Candelaria, Crta del Rosario s/n, Santa Cruz de Tenerife, 38010 Spain; Intensive Care Unit, Hospital General La Palma, Buenavista de Arriba s/n, Breña Alta, La Palma 38713 Spain; Intensive Care Unit, Hospital Insular, Plaza Dr. Pasteur s/n, Las Palmas de Gran Canaria, 35016 Spain; Intensive Care Unit, Hospital Universitario Dr. Negrín, Barranco de la Ballena s/n, Las Palmas de Gran Canaria, 35010 Spain; Intensive Care Unit, Hospital Clínico Universitario de Valencia, Avda. Blasco Ibáñez n°17-19, Valencia, 46004 Spain; Research Unit, Hospital Universitario de Canarias, Ofra, s/n. La Laguna, 38320 Santa Cruz de Tenerife, Spain; Laboratory Deparment, Hospital Universitario de Canarias, Ofra, s/n. La Laguna, 38320 Santa Cruz de Tenerife, Spain; Atherosclerosis Research Laboratory, CIMA-University of Navarra, Avda Pío XII n°55, Pamplona, 31008 Spain

**Keywords:** TIMP-1, Ischemic stroke, Patients, Mortality, Injury

## Abstract

**Background:**

In the last years, circulating matrix metalloproteinases (MMP)-9 levels have been associated with functional outcome in ischemic stroke patients. However the prognostic value of circulating levels of tissue inhibitor of matrix metalloproteinases (TIMP)-1 and MMP-10 in functional outcome of ischemic stroke patients has been scarcely studied. In addition, to our knowledge, serum MMP-9, MMP-10 and TIMP-1 levels in patients with malignant middle cerebral artery infarction (MMCAI) for mortality prediction have not been studied, and these were the objectives of this study.

**Methods:**

This was a multicenter, observational and prospective study carried out in six Spanish Intensive Care Units. We included patients with severe MMCAI defined as Glasgow Coma Scale (GCS) lower than 9. We measured circulating levels of MMP-9, MMP-10, TIMP-1, in 50 patients with severe MMCAI at diagnosis and in 50 healthy subjects. Endpoint was 30-day mortality.

**Results:**

Patients with severe MMCAI showed higher serum levels of MMP-9 (p = 0.001), MMP-10 (p < 0.001), and TIMP-1 (p = 0.02) than healthy subjects. Non-surviving MMCAI patients (n = 26) compared to survivor ones (n = 24) showed higher circulating levels of TIMP-1 (p < 0.001), MMP-10 (p = 0.02) and PAI-1(p = 0.02), and lower MMP-9 levels (p = 0.04). Multiple binomial logistic regression analysis showed that serum TIMP-1 levels > 239 ng/mL are associated with 30-day mortality (OR = 5.82; 95 % CI = 1.37-24.73; P = 0.02) controlling for GCS and age. The area under the curve for TIMP-1 as predictor of 30-day mortality was 0.81 (95 % CI = 0.67-0.91; P < 0.001). We found an association between circulating levels of TIMP-1 and MMP-10 (rho = 0.45; P = 0.001), plasminogen activator inhibitor (PAI)-1 (rho = 0.53; P < 0.001), and tumor necrosis factor (TNF)-alpha (rho = 0.70; P < 0.001).

**Conclusions:**

The most relevant and new findings of our study, were that serum TIMP-1 levels in MMCAI patients were associated with mortality, and could be used as a prognostic biomarker of mortality in MMCAI patients.

## Background

Ischemic stroke is an important cause of disability, mortality and resources consume [1]. Matrix metalloproteinases (MMPs) are implicated in degradation and remodelling of the extracellular matrix (ECM). That family of zinc-containing endoproteinases can be classified according to the substrate specificity as collagenases (MMP-1, −8 and −13), gelatinases (MMP-2 and −9), stromelysins (MMP-3, −10, −11), matrilysins (MMP-7), elastases (MMP-12) and membrane-type (MT-MMPs, MMP-14, −15, −16 and −17). MMP activity is regulated by specific tissue inhibitors of matrix metalloproteinases (TIMPs). MMPs are involved in physiological functions such as morphogenesis, menstrual cycle, tissue remodelling and angiogenesis; and also in some diseases with abnormal ECM turnover, such as arthritis, sepsis, tumour invasion and atherosclerosis [2–7].

In the last years, MMPs have been found to play a role in cerebral ischemia [8–10]. In some studies higher circulating MMP-9 levels were found in ischemic stroke patients than in controls [11–15], and in ischemic stroke patients with worse functional outcome [11–19]. However the prognostic value of circulating levels of TIMP-1 [20] and MMP-10 [21] in functional outcome of ischemic stroke patients has been scarcely studied. Circulating TIMP-1 levels have been associated with poor prognosis in a community-based cohort of elderly men risk [22], patients with coronary artery disease [23], and in different cancer types, such as lung [24] breast [25] colorectal [26] and gastric cancer [27]. There have been found higher TIMP-1 concentrations in infarcted brain tissue compared to healthy cerebral areas [28], higher expression of TIMP-1 in monocytes of ischemic stroke patients than in healthy controls [29], and higher circulating TIMP-1 levels in ischemic stroke patients than in healthy controls [30–33]. In addition, there has been found an association between serum TIMP-1 levels and mortality in patients with severe trauma brain injury [34].

To our knowledge, serum MMP-9, MMP-10 and TIMP-1 levels in patients with malignant middle cerebral artery infarction (MMCAI) for mortality prediction have not been studied, and these were the objectives of this study.

## Methods

### Design and subjects

This is a multicenter, observational, prospective study carried out in 6 Intensive Care Units of Spain. The study was approved by the Institutional Review Board of the 6 participant hospitals: Hospital Universitario de Canarias (La Laguna, Santa Cruz de Tenerife, Spain), Hospital Universitario Nuestra Señora de Candelaria (Santa Cruz de Tenerife, Spain), Hospital General de La Palma (La Palma, Spain), Hospital Clínico Universitario de Valencia (Valencia, Spain), Hospital Insular (Las Palmas de Gran Canaria, Spain), Hospital Universitario Dr. Negrín (Las Palmas de Gran Canaria, Spain). The written informed consent from the patients or from their legal guardians was obtained.

We included 50 patients with severe MMCAI and 50 healthy volunteer control subjects. Severity of MMCAI was classified according to Glasgow Coma Scale (GCS) [35], and severe was defined as GCS ≤ 8. Exclusion criteria were: age less than 18 years, inflammatory or malignant disease.

### Variables recorded

The following variables were recorded for each patient: sex, fibrinolityc therapy, decompressive craniectomy, age, temperature, sodium, glycemia, leukocytes, pressure of arterial oxygen (PaO2), PaO2/ pressure of arterial oxygen/fraction inspired oxygen (FI0_2_) ratio, bilirubin, creatinine, hemoglobin, GCS, lactic acid, platelets, international normalized ratio (INR), activated partial thromboplastin time (aPTT), fibrinogen, Acute Physiology and Chronic Health Evaluation II (APACHE II) score [36]. The end-point of the study was 30-days mortality.

### Blood sample collection

Blood samples of 50 patients with severe MMCAI were collected at the moment of the diagnosis and of 50 controls to measure the concentrations of MMP-9, MMP-10, TIMP-1, tumor necrosis factor (TNF)-alpha, and plasminogen activator inhibitor (PAI)-1. To avoid the possible dispersion of serum level results, all the samples were processed at same time and in the same laboratory, at the end of the recruitment process.

### Determination of serum MMP-9, MMP-10, TIMP-1 and TNF-alpha levels

Serum separator tubes were used to determine serum MMP-9, MMP-10, TIMP-1 and TNF-alpha levels. Venous blood samples were taken and centrifuged within 30 min at 1000 g for 15 min, and the serum was removed and frozen at −80 °C until measurement.

MMP-9, MMP-10 and TIMP-1 assays were performed at the Atherosclerosis Research Laboratory of CIMA-University of Navarra (Pamplona, Spain) and were assayed by specific ELISAs (Quantikine®, R&D Systems, Abingdon, United Kingdom) according to the manufacturer's instructions with a serum dilution of 1:80, 1:2 and 1:100 respectively. The interassay coefficients of variation (CV) were <8 % (n = 20) and detection limit for the assays were 0.31 ng/ml, 78.1 pg/ml and 0.15 ng/ml respectively.

TNF-alpha serum levels were measured in the Laboratory Deparment of the Hospital Universitario de Canarias (La Laguna, Santa Cruz de Tenerife, Spain) by a solid-phase, chemiluminiscents immunometrics assays kit (Immulite®, Siemens Healthcare Diagnostics Products, Llanberis, United Kingdom); and the interassays CV was <6.5 % (n = 20) and detection limit for the assay was 1.7 pg/mL.

### Determination of plasma PAI-1 levels

Venous blood samples were collected in citrate collected plasma tubes and centrifugedwithin 30 min at 1000*g for 15 min. The plasma was removed and frozen at −80 °C until measurement. PAI-1 assay was performed at the Laboratory Department of the Hospital Universitario de Canarias (La Laguna, Santa Cruz de Tenerife, Spain). PAI-1 antigen levels were assayed by specific ELISA (Imubind Plasma PAI-1 American Diagnostica, Inc, Stanford, CT, USA). The interassay CV of PAI-1 assay was <5 % (n = 20) and detection limits was 1 ng/mL.

### Statistical methods

Continuous variables are reported as medians and interquartile ranges. Categorical variables are reported as frequencies and percentages. Comparisons of continuous variables between groups were carried out using Wilcoxon-Mann–Whitney test. Comparisons between groups on categorical variables were carried out with chi-square test.

Multiple binomial logistic regression analysis was applied to determine the independent contribution of TIMP-1 on 30-day mortality, controlling for GCS and age. Odds Ratio and 95 % confidence intervals were calculated as measurement of the clinical impact of the predictor variables.

Receiver operating characteristic (ROC) analysis was carried out to determine the goodness-of-fit of the of serum TIMP-1 levels to predict 30-day mortality. Kaplan-Meier analysis of survival at 30 days and comparisons by log-rank test were carried out using serum TIMP-1 levels lower/higher than 239 ng/mL as the independent variable and survival at 30 days as the dependent variable. The association between continuous variables was carried out using Spearman’s rank correlation coefficient. A *P* value of less than 0.05 was considered statistically significant. Statistical analyses were performed with SPSS 17.0 (SPSS Inc., Chicago, IL, USA) and NCSS 2000 (Kaysville, Utah) and LogXact 4.1, (Cytel Co., Cambridge, MA).

## Results

Patients with severe MMCAI showed higher serum levels of MMP-9, MMP-10 and TIMP-1 than healthy subjects (Table 1).

**Table 1:** Characteristics of healthy controls and patients with severe MMCAI

	Healthy controls (n = 50)	Patients (n = 50)	p-value
Gender female – n (%)	13 (26.0 %)	17 (34 %)	0.51
Age (years) - median (p 25–75)	57 (50–63)	60 (51–69)	0.11
TIMP-1 (ng/mL) - median (p 25–75)	226 (213–241)	261 (199–387)	0.02
MMP-9 (ng/mL) - median (p 25–75)	498 (350–735)	749 (488–1200)	0.001
MMP-10 (pg/mL) - median (p 25–75)	466 (288–614)	1027 (556–1409)	<0.001

MMP = matrix metalloproteinase; TIMP = tissue inhibitor of matrix metalloproteinases

We found that non-surviving MMCAI patients (n = 26) compared to survivors ones (n = 24) showed higher circulating levels of MMP-10, TIMP-1, PAI-1 and TNF-alpha, and lower MMP-9 levels (Table 2).

**Table 2:** Clinical and biochemical characteristics of survivor and non-survivor MMCAI patients

	Survivors (n = 24)	Non-survivors (n = 26)	P value
Gender female – n (%)	8 (33.3)	9 (34.6)	0.99
Decompressive craniectomy – n (%)	7 (29.2)	5 (19.2)	0.51
Age (years) - median (p 25–75)	47 (32–67)	66 (45–76)	0.14
Temperature (°C) - median (p 25–75)	36.5 (35.7-37.0)	37.0 (35.7-37.8)	0.26
Sodium (mEq/L)- median (p 25–75)	140 (138–145)	140 (137–146)	0.91
Glycemia (g/dL) - median (p 25–75)	133 (105–170)	135 (110–154)	0.92
Leukocytes-median*10^3^/mm^3^ (p 25–75)	12.8 (9.8-16.9)	14.4 (11.9-21.9)	0.49
PaO2 (mmHg) - median (p 25–75)	110 (101–194)	104 (85–139)	0.10
PaO2/FI0_2_ ratio - median (p 25–75)	246 (192–327)	248 (175–320)	0.41
Bilirubin (mg/dl) - median (p 25–75)	0.50 (0.38-0.90)	0.53 (0.30-1.20)	0.76
Creatinine (mg/dl) - median (p 25–75)	0.80 (0.60-1.10)	1.01 (0.85-1.45)	0.052
Hemoglobin (g/dL) - median (p 25–75)	12.0 (11.3-13.8)	12.0 (11.0-15.1)	0.92
GCS score - median (p 25–75)	7 (6–8)	6 (4–8)	0.10
Lactic acid (mmol/L)-median (p 25–75)	1.25 (0.93-1.68)	1.50 (1.01-3.15)	0.08
Platelets - median*10^3^/mm^3^ (p 25–75)	227(183–308)	152 (123–190)	0.003
INR - median (p 25–75)	1.07 (1.01-1.20)	1.20 (1.07-1.48)	0.16
aPTT (seconds) - median (p 25–75)	28 (25–29)	26 (25–33)	0.96
Fibrinogen (mg/dl) - median (p 25–75)	440 (335–494)	409 (322–598)	0.71
APACHE-II score - median (p 25–75)	20 (16–25)	22 (19–29)	0.14
MMP-9 (ng/mL) - median (p 25–75)	963 (731–1218)	672 (384–1088)	0.04
MMP-10 (pg/mL) - median (p 25–75)	785 (550–1114)	1264 (608–1759)	0.02
TIMP-1 (ng/mL) - median (p 25–75)	204 (172–264)	343 (240–493)	<0.001
PAI-1 (ng/mL) - median (p 25–75)	24.0 (19.3-40.8)	51.5 (28.3-95.3)	0.02
TNF-alpha (pg/mL) - median (p 25–75)	9.25 (9.02-10.63)	12.95 (10.03-15.08)	0.01

P 25-75 = percentile 25^th^-75^th^; PaO_2_ = pressure of arterial oxygen/fraction inspired oxygen; FIO_2_ = pressure of arterial oxygen/fraction inspired oxygen; GCS = Glasgow Coma Scale; ISS = Injury Severity Score; INR = international normalized ratio; aPTT = activated partial thromboplastin time; APACHE II = Acute Physiology and Chronic Health Evaluation; MMP = matrix metalloproteinase; TIMP = tissue inhibitor of matrix metalloproteinases; PAI = plasminogen activator inhibitor; TNF = tumor necrosis factor

Multiple binomial logistic regression analysis showed that serum TIMP-1 levels > 239 ng/mL are associated with 30-day mortality (OR = 5.82; 95 % CI = 1.37-24.73; P = 0.02) controlling for GCS and age (Table 3).

**Table 3:** Multiple binomial logistic regression analysis to predict 30-day mortality

Variable	Odds Ratio	95 % Confidence Interval	*P*
TIMP-1 > 239 ng/mmL	5.82	1.37-24-73	0.02
GCS score	0.79	0.56-1.12	0.19
Age (years)	1.01	0.95-1.07	0.81

**Fig. 1:**
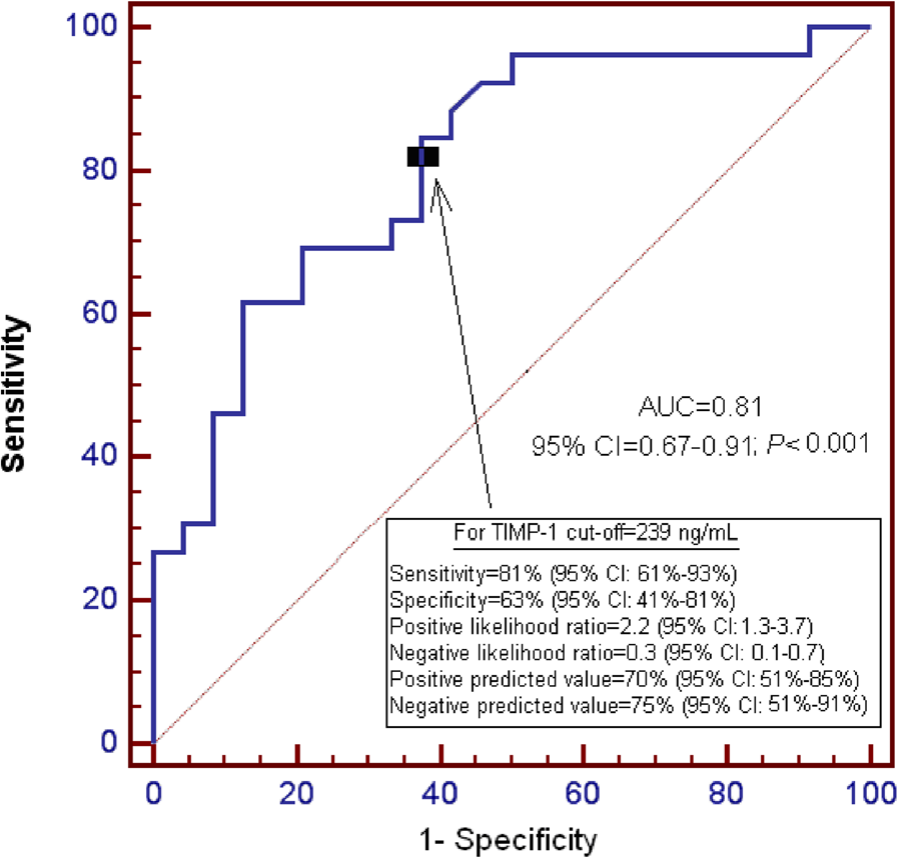
Receiver operation characteristic analysis using TIMP-1 serum levels as predictor of mortality at 30 days

The area under the curve (AUC) for TIMP-1 as predictor of 30-day mortality was 0.81 (95 % CI = 0.67-0.91; P < 0.001) (Fig. 1).

**Fig. 2:**
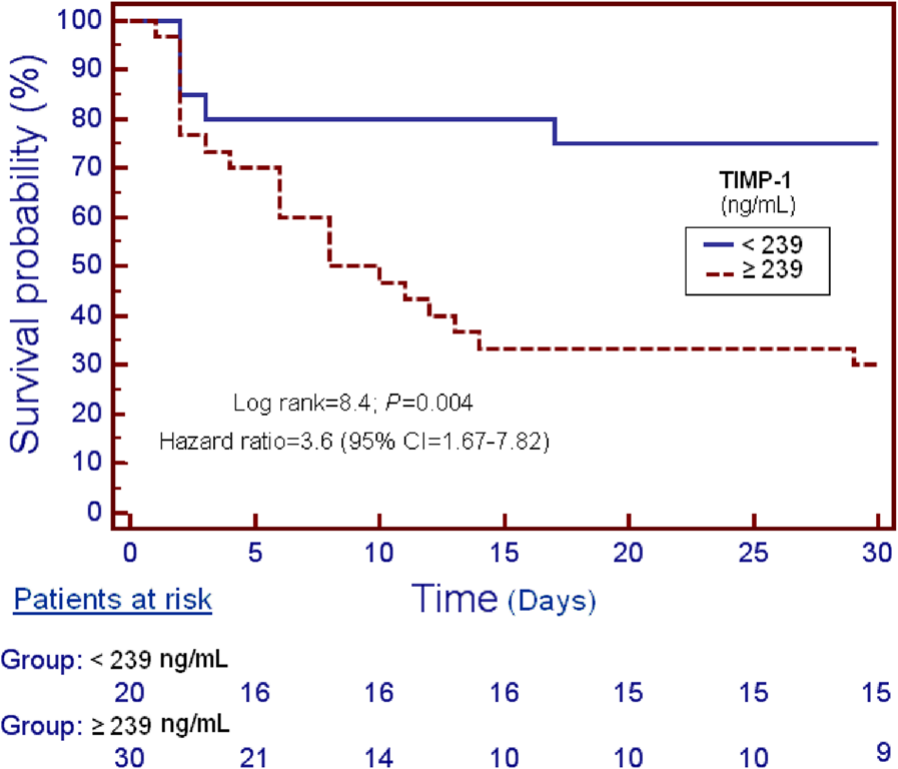
Survival curves at 30 days using TIMP-1 serum levels higher or lower than 239 ng/mL

Survival analysis showed that patients with serum TIMP-1 higher than 239 ng/mL presented higher 30-day mortality than patients with lower levels (Hazard ratio = 3.6; 95 % CI = 1.67-7.82; *P =* 0.004) (Fig. 2).

We found an association between circulating levels of TIMP-1 and MMP-10 (rho = 0.45; P = 0.001), PAI-1 (rho = 0.53; P < 0.001), and TNF-alpha (rho = 0.75; P < 0.001).

## Discussion

The novel findings of our study were the following: a) non-surviving severe MMCAI patients had higher serum TIMP-1 and MMP-10 levels than surviving patients; b) there is an association between circulating levels of TIMP-1, PAI-1, and TNF-alpha in patients with severe MMCAI; c) serum TIMP-1 levels could be used as prognostic biomarker in patients with severe MMCAI.

We found that patients with severe MMCAI showed higher serum levels of MMP-9, MMP-10 and TIMP-1 than healthy subjects. Previously there were found higher circulating levels of MMP-9 [11–15] and MMP-10 [21] and TIMP-1 [30–33] in ischemic stroke patients than in controls. In addition, there have been found higher production of TIMP-1 in infarcted brain tissue compared to healthy brain areas [28], and higher expression of TIMP-1 in monocytes of ischemic stroke patients than in healthy controls [29].

In addition, we found higher circulating MMP-10 and TIMP-1 levels, and lower circulating MMP-9 levels in non-surviving severe MMCAI patients than in surviving patients. The findings in respect to MMP-10 are in consonance with a previous study showing an association between serum MMP-10 and functional outcome in ischemic stroke patients [21]; however, in our current study, we found for the first time higher serum MMP-10 in non-surviving than in surviving MMCAI patients.

Our findings, showing higher TIMP-1 levels in non-surviving severe MMCAI patients than in surviving patients, could be in agreement with the results of other previous study [20]. A relationship between plasma TIMP-1 levels at 7 days of clinical ischemic with neurological clinical outcome has been demonstrated [20]. Then, another new finding of our study were those higher serum TIMP-1 levels at moment of severe MMCAI diagnosis in non-survivor than in survivor patients. Previously, there have been found an association between circulating TIMP-1 levels and poor prognosis in elderly men [22], patients with coronary artery disease [23], patients with different types of cancer [24–27], and patients with severe trauma brain injury [34].

On the other hand, the results regarding to circulating MMP-9 levels are in contradiction with those previously published reporting a poor functional outcome with high circulating MMP-9 levels [11–19].

Another interesting new findings of our study were the association between serum TIMP-1 levels and mortality in logistic regression analysis, and the mortality prediction of circulating TIMP-1 levels according to the ROC analysis. These findings agree with the results of a previous study by our team in patients with severe trauma brain injury [34].

The pathophysiological role of circulating TIMP-1 levels in MMCAI patients is still unknown. It is possible that the increased levels in these patients may be due to an increase of circulating MMP-9 and MMP-2 levels, in order to maintain the balance between proteases and inhibitors. Interestingly, we report for the first time an association between circulating levels of TIMP-1 and MMP-10, PAI-1, and TNF-alpha in patients with severe MMCAI patients. Previously higher circulating levels of PAI-1 [37, 38] and TNF-alpha [21] were found in ischemic stroke patients with poor functional outcome. Taken together, these data suggest that TIMP-1 levels could play a role in the pathophysiology of MMCAI. It is possible that increased serum TIMP-1 levels in non-survivors TBI patients is not the cause of death in these patients, rather a biomarker associated with mortality.

Some limitations of our study should be recognized. First, we did not report data about the evolution of TIMPs and MMPs on the time to describe the evolution in non-surviving and surviving TBI patients. Second, the determination of other MMPs and TIMPs would be desirable. Third, the assessment of other inflammatory cytokines and coagulation biomarker could be interesting. Four, there is overlap of serum TIMP-1 levels between dead and alive patients at 30 days; thus, the sole use of serum TIMP-1 levels to predict 30-day survival in MMCAI patients should be taken with caution. However, we think that the findings of our study (reporting for the first time an association between TIMP-1 and mortality in MMCAI patients) could open the interest for research about TIMP-1 in MMCAI patients.

The administration of modulators of MMP activity have showed a beneficial effect in rat ischemic stroke models reducing the expression of MMPs, blood–brain barrier leakage, volumen infarction, neurological dysfunction and mortality [39–44]. Thus, from a therapeutic perspective, MMP activity modulators levels could be used as a new class of drugs for the treatment of patients with severe ischemic stroke.

## Conclusions

The most relevant and new findings of our study, were that serum TIMP-1 levels in MMCAI patients were associated with mortality, and could be used as a prognostic biomarker of mortality in MMCAI patients.

## Abbreviations

MMP: Matrix metalloproteinases
TIMP: Tissue inhibitor of matrix metalloproteinases
ICU: Intensive Care Unit
PaO_2_: Pressure of arterial oxygen/fraction inspired oxygen
FIO_2_: Pressure of arterial oxygen/fraction inspired oxygen
GCS: Glasgow Coma Scale
INR: International normalized ratio
aPTT: activated partial thromboplastin time
APACHE II: Acute Physiology and Chronic Health Evaluation
PAI: Plasminogen activator inhibitor
TNF: Tumor necrosis factor
